# The Movement Cure

**Published:** 1860-10

**Authors:** 


### ARTICLE IV.

### *The Movement Cure.*

Human ingenuity exhausts itself to supply man's real wants less effectually than it does the supposititious demands of his infirmities and his imaginary evils.

In the time of Galen and Hippocrates the resort to the actually known remedies was less than to the fabulous and the unknown. Some wondrous insect was swallowed or decocted to cure a broken leg or diseased liver. Herbs were invested with magical power and sought after by emperors and kings to regenerate the morals of their subjects. Even in Elizabeth's time grave physicians lectured upon the heaven gifted influence of combinations as absurd as repulsive, finding the lord and the boor out of number, who had been blessed by their healing influences.

Mazzini and his loved and loving queen trusted and feared their absolute quack whilst they abused and suspected their truly honest and learned doctor. Homeopathy found crowds of silly idolators, who obeyed and trusted, swallowed and prayed, cured by nothing, in worshipping the little pellicle and the vulgar nonsense of Hahnemann. Later, steady England gave birth to the *brandy* and *salt* cure, built a fine establishment to remedy evils by rubbing in, what had been produced by pouring in, with all other evils poor flesh inherits. Hydropathy drew thousands to have their wits renewed by constant washings. Sleeping in water, walking in water, living with vapors and water perpetually, was believed capable of anything however absurd and ridiculous the fact. A dry cough—wet sheet, a broken limb—wet sheet, chronic pneumonia—wet sheet, gout—wet sheet, a broken conscience—a wet sheet, a broken heart—a wet sheet. The babe in its cradle with its mother's tears, the feeble old man, dropping kindly into the grave, were all subjects for the wet sheet—yes, and was believed it would cure your uncle dead an hundred years, or subjects quite as reasonable. Rochester wizzards', Clairvoyants' and Spiritualists' power have all been addressed and performed their wonders, had their infancy of promise, youth of growth and indulgence—old age of puling decay and death, their victims buried by the graves of their fathers, and their memories, like their ghosts, invisible and unfelt.

Human nature and human ingenuity, true to their instincts, on the one hand, call for something new, and on the other hand, most lugubriously supply the call, by sending the *Movement Cure*, from Switzerland or Germany, the birth-place of many monsters, perhaps the last, the least, for it simply advocates the sac-cloth and ash cure, of pounding the diseased part until it whips it into subjection.

Dear reader, you may have the indigestion, as I take you for a dentist, well you are now to be cured by taking a *padded paddle*, on one side soft, the other hard, and you are to belabor your stomach until its sore complaints are silenced, and to aid it in this worthy purpose you are to beat the rest of your body until induration relieves you of all trouble, in your becoming like a rhinoceros or alligator to all applied forces. There is something extremely pleasant about this coming nonsense. *This new god of the hypochondriacs*, is *destined to become*, as by comparison, *very popular*, for it gives one a hope that justice may be done occasionally, blindly though it be, by having your enemies soundly beaten at times, for all our enemies are fools and will require the *movement cure* to aid them. One thing is to be feared, that all may wish to become doctors of it, our brotherhood consequently deriving considerable relief, from which we gain some hope and consolation.

We said extremely pleasant this movement cure, fancy a patient with the rheumatism or gout going through a dose of *movement*. He is belabored and boxed after a perfect system of hardest blows to sorest parts, no matter how they wince or groan, blow follows blow until they actually yell with pain and exhaustion. Seriously, we think it an admirable remedy for the ills of the country of its birth, life's lassitudes are so easily borne by the German, and his languid dullness makes him a constitutional dreamer, this *movement cure* would kindly awaken him from his metaphysics, dispel the vapor which surrounds his mental vision, and justly punish him for introducing the silly, wondrous pellicle.

Something sagaciously practical in beating our poor bodies for their physical misdemeanors, but we can hardly overlook the man who is always saying *yaw*, for introducing corporeal punishment for the inherited evils of our forefathers, for thus the just perhaps may receive punishment for the unjust. The consumptive subject instead of passing from time to eternity in peaceful quiet, must thus pound his poor breast to atone for the errors of his progenitors.

Is it possible that the history of this year is to be burdened by the silly tradition of this irresistibly comic proposition? We look around in hopes of some means to pass opinion from, in the negative, but alas when we see mankind in herds, like cattle, grazing down the opposition to their monstrosities in one section, still growing, emigrating to another; societies, springing up confessedly in opposition to the purest conservatism of domestic and political life; vagaries assuming systematic existence, we are compelled to admit, that human nature in its abstract points, is the same now as hundreds of years ago, when a cock chaffer was thought to possess the power of reinvigorating the germ of life, so now thumping the body will doubtless have many attachés, who will believe it to be the last scientific discovery.

